# Diversity of opisthokont septin proteins reveals structural constraints and conserved motifs

**DOI:** 10.1186/s12862-018-1297-8

**Published:** 2019-01-07

**Authors:** Benjamin Auxier, Jaclyn Dee, Mary L. Berbee, Michelle Momany

**Affiliations:** 1 Department of Botany, University of British Columbia, Vancouver, Canada; 2 Fungal Biology Group and Plant Biology Department, University of Georgia, Athens, USA; 3 current address: Laboratory of Genetics, Wageningen University and Research, P.O. Box 16, 6700AA, Wageningen, The Netherlands

**Keywords:** Septin, Subunit, Modelling, Protein-protein interaction, Opisthokont, Evolution, Gene tree-species tree reconciliation, Ancestral state reconstruction

## Abstract

**Background:**

Septins are cytoskeletal proteins important in cell division and in establishing and maintaining cell polarity. Although septins are found in various eukaryotes, septin genes had the richest history of duplication and diversification in the animals, fungi and protists that comprise opisthokonts. Opisthokont septin paralogs encode modular proteins that assemble into heteropolymeric higher order structures. The heteropolymers can create physical barriers to diffusion or serve as scaffolds organizing other morphogenetic proteins. How the paralogous septin modules interact to form heteropolymers is still unclear. Through comparative analyses, we hoped to clarify the evolutionary origin of septin diversity and to suggest which amino acid residues were responsible for subunit binding specificity.

**Results:**

Here we take advantage of newly sequenced genomes to reconcile septin gene trees with a species phylogeny from 22 animals, fungi and protists. Our phylogenetic analysis divided 120 septins representing the 22 taxa into seven clades (Groups) of paralogs. Suggesting that septin genes duplicated early in opisthokont evolution, animal and fungal lineages share septin Groups 1A, 4 and possibly also 1B and 2. Group 5 septins were present in fungi but not in animals and whether they were present in the opisthokont ancestor was unclear. Protein homology folding showed that previously identified conserved septin motifs were all located near interface regions between the adjacent septin monomers. We found specific interface residues associated with each septin Group that are candidates for providing subunit binding specificity.

**Conclusions:**

This work reveals that duplication of septin genes began in an ancestral opisthokont more than a billion years ago and continued through the diversification of animals and fungi. Evidence for evolutionary conservation of ~ 49 interface residues will inform mutagenesis experiments and lead to improved understanding of the rules guiding septin heteropolymer formation and from there, to improved understanding of development of form in animals and fungi.

**Electronic supplementary material:**

The online version of this article (10.1186/s12862-018-1297-8) contains supplementary material, which is available to authorized users.

## Background

From their common unicellular ancestor ~ 1.3 billion years ago, opisthokonts, the clade uniting animals and fungi inherited a core set of genes, which through duplications, deletions and other modifications gave rise to an astounding range of morphological and physiological diversity [1]. Septin genes are among those that appear to have expanded during opisthokont diversification. Given their important roles in morphogenesis, septin proteins may have contributed to the evolution of opisthokont complexity and diversity. Alongside the better known proteins that form actin filaments, intermediate filaments, and microtubules, septins assemble into filaments or rings that constitute part of the cytoskeleton [2]. In both animals and fungi, septins form physical barriers to diffusion and also anchor proteins to substrates such as the plasma membrane and endoplasmic reticulum [3, 4]. In animals, septins are involved in cell division, and localize to the plasma membrane during cytokinesis [3]. Perturbation of septins is associated with many diseases [2, 5]. In fungi, septins are involved in cell division and in determining morphology [6, 7]. In addition to morphology, septins are required for virulence in many fungal pathogens of plants and animals [8–10].

**Fig. 1:**
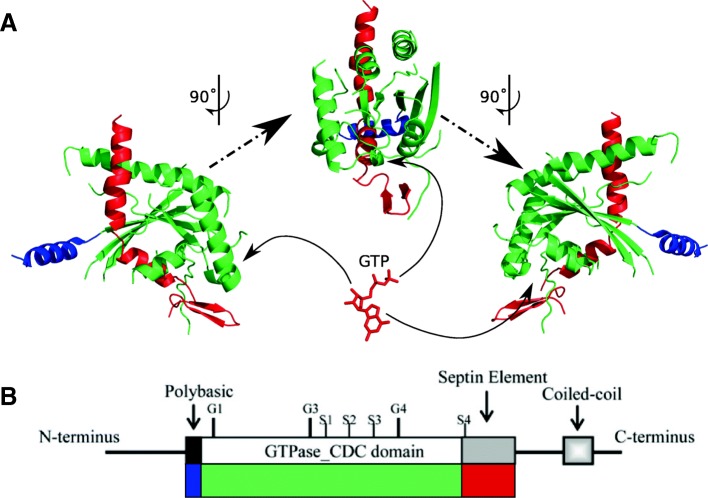
– Organization of a septin protein. **a**) Three-dimensional crystal structure of human septin Sept2 (PDB: 2QA5) produced with pymol showing three orientations, each rotated 90°. Position of GTP binding is shown by solid arrow. Structures are coloured according to regions in **b**. Note that C-terminal extensions are not resolved in the crystal structure. **b**) Linear representation of a septin protein, indicating the arrangements of the major septin elements as described in the text. **b**) Reproduced with permission from Pan et al., [15]

Structurally, septin proteins (Fig. 1) consist of an amino-terminal extension (NTE) that is highly variable, which borders a poly-basic (PB) domain. The PB domain is presumed to interact with membranes by binding phosphoinositides [11, 12]. The center of the septin protein sequence is a highly-conserved Ras-type GTPase. The role of the GTPase is thought to be structural rather than catalytic due to its extremely slow hydrolysis of GTP [13, 14]. Carboxy-terminal to the GTPase is a septin unique element (SUE) that has a largely unknown role, although some studies have suggested involvement in interface binding [12]. The carboxy-terminal extension (CTE) of the protein is, like the NTE, highly variable. The CTE is predicted to form coiled-coils in many opisthokont septins, which contribute to forming septin higher order structures (HOS) [15]. To form HOS, individual septins first form heterooligomers by interacting through their G- and NC-interfaces, and these heterooligomers interact with each other end-to-end to form filaments and laterally via CTEs to from bundles [16, 17]. Although different types of heteropolymers can co-exist in a cell, the order of monomers is not random and instead shows consistencies across all the septin heterooligomer types [18, 19]. Human septin monomers and their heterooligomers have been analyzed by crystallography and transmission electron microscopy, leading to an increasingly sophisticated understanding of the basis for monomer assembly at the level of interactions of interface amino acid residues [12, 20]. With available crystal structures of the human septins, it becomes possible to model the three-dimensional structure of other orthologous opisthokont sequences. This opens the door to recognizing interactions between conserved amino acid residues in aligned opisthokont septins. These interactions then direct septin heterooligomer self-assembly.

New whole genome sequences from unicellular relatives of animals and from non-filamentous fungi provide opportunities to relate patterns of gene evolution and morphological complexity to increasingly sophisticated organismal phylogenies. Our first aim here was to analyze patterns of septin gene duplication and loss in the context of organismal evolution. Our second aim was to use protein homology folding to identify conserved amino acid residues with potential roles in binding between subunits, thereby contributing to an understanding of the rules governing the assembly of septin heteropolymers.

## Methods

### Septin searches and coiled-coil domain prediction

To identify opisthokont septins, we downloaded the predicted proteomes of 22 taxa (Additional file 1: Table S1). We searched these using PSI-BLAST with *Saccharomyces cerevisiae* CDC3 (NP_013418.2) as the initial query and an e-value cutoff of 0.001. Three iterations of PSI-BLAST recovered all known septins from model organisms *Aspergillus, Drosophila,* and *Saccharomyces*. As an alternative search strategy we also used HMMER [21] with a previous alignment as a search profile [15]. As the GTPase domain has sequence similarity with many other proteins, we used the Conserved Domain Database (CDD) [22] and a custom script to retain proteins with recognized domains “P-Loop_NTPase”, “CDC3”, or “CDC_septin”; or else with at least two of three G boxes (G1, G3, G4) in the GTPase domain without conflicting domains such as “LysM” or “DNA_binding”. These alternative criteria allowed us to include *S. cerevisiae* CDC3 even through CDD did not recognize three G boxes in the gene, as well as *A. nidulans* AspE for which CDD did not recognize a CDC3 or CDC_Septin domain. Filtering retained 120 septins. As the criteria were based on previously known septins, we cannot exclude that we may have overlooked novel diversity. To assess septins in the opisthokont ancestor, we searched the genomes of *Thecamonas trahens* and *Lenisia limosa*, two members of Apusozoa, the sister group to opisthokonts [23], but no septin sequences were recovered. The 120 filtered proteins were aligned with COBALT [24] with default settings, and the alignment was visualized with Mesquite [25]. To predict coiled-coil domains known to be involved in septin interactions, we used the MarCoil webserver with the 120 proteins [26].

### Phylogenetic analysis

We processed the septin alignment using Aliscore and Alicut (window size, 12) to remove areas where positional homology was doubtful, retaining 344 sites [27, 28]. Using ProtTest3 with AIC [29], we chose LG + gamma + invariant sites as an appropriate model of evolution for subsequent analyses. Using RAxML v8.0 through the CIPRES portal [30, 31], we performed 2000 maximum likelihood searches and then 456 bootstrap replicates (a sufficient number based on -autoMRE option) [32]. We also performed the same RAxML analysis but with partitioning of the 49 interacting sites (detailed below). We also performed a Bayesian analysis with MrBayes v3.2.4 using two independent runs of 8 chains, modifying the heating parameter to 0.06 to increase the swap frequency and running the analysis for 200 million generations [33]. We used TRACER to select a burn-in of 50%. After excluding the burn-in generations, the average split frequency was below 0.01 and all parameter effective sample sizes were over 200 [34].

**Fig. 2:**
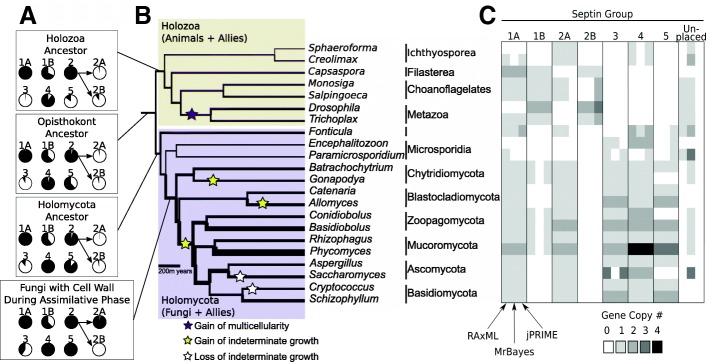
Analysis of early-diverging lineages provides evidence of ancestral septin duplications. **a**) Shaded area of pie charts indicates the proportional likelihood that a specific ancestor had a member of a septin group, when reconstructed under maximum likelihood. As Group 2 may or may not be monophyletic, reconstructions resulting from these two alternatives are illustrated. **b**) Gene copy number reconstructed within the species phylogeny. Branch thickness represents the average NOTUNG inferred number of septin gene copies based on the jPRIME septin gene phylogeny. Stars indicate a change in morphology of organisms in a lineage. Note: This species phylogeny was used to guide the jPRIME analysis. **c**) Cell shading indicates copy numbers of genes representing each septin group, classified by each of three phylogenetic methods. Classification of septins from an organism sometimes differed depending on the analysis method, reflecting uncertainty in phylogenetic placement of divergent sequences

We also performed a jPRIME analysis to increase phylogenetic resolution by using an ultrametric organismal phylogeny as an informative prior to parameterize gene duplication and loss rates [35, 36]. For the required organismal phylogeny we used a topology from Torruella et al. [37, 38] (Fig. 2). Because not all of our taxa were included in Torruella et al.’s analysis, we re-estimated all branch lengths using a new set of genomic data for all species. With OrthoFinder we identified orthologous gene groups for the 22 taxa [39], aligning orthologs with MAFFT with the –auto setting and concatenating them with FasconCAT [40]. With ProtTest3, we selected the LG model of evolution for the concatenated alignment. We used RAxML v8.0 to infer branch lengths for the Torruella et al. topology. We used the Turner-Nash method and cross-validation to select a smoothing value of 14 with r8s [41] to transform the tree to be ultrametric. We constrained the age of the basal node of the opisthokonts to 1350 million years, within the range estimated by Parfrey et al. [42].

We then ran two independent jPRIME analyses of the aligned septin genes for 50 million generations each using the ultrametric organismal tree, and applying RWTY [43] to confirm that the split frequency standard deviation was below 0.01. TRACER showed that after discarding the first 50% of the trees, the estimated sample sizes were over 200 for all parameters except for the gene duplication rate and the model of molecular evolution, which were both over 100. To summarize the jPRIME results with a Maximum Clade Credibility Topology, we applied SumTrees from the DendroPy Python library [44]. We used the packages ape and ggtree [45, 46] in R 1.0.143 to visualize the phylogenies. We rooted the septin phylogenies with the clade of Group 3 and 5 for reasons discussed below.

### Septin gene tree reconciliation with the animal and fungal organismal phylogeny and septin ancestral state reconstruction

To reconstruct septin gene duplications and losses along the opisthokont phylogeny, we reconciled the jPRIME septin phylogeny with the species tree using NOTUNG v2.8.1.7 [47]. We allowed rearrangements of the septin phylogeny for nodes with a posterior probability below 0.90, which tends to minimize the overall number of duplications and losses. This resulted in 2 equally parsimonious reconciliations, the average of which we indicate by the thickness of the branches of a species tree. To track the origin of septin groups within the organismal phylogeny, we reconstructed ancestral states with Mesquite v3.2. To designate Group identity, we anchored the largest monophyletic group possible around *Aspergillus* or *Drosophila* septins (excluding NP_724659 as it did not consistently group with other two *Drosophila* Group 2 septins) (Additional file 2: Figure S1-3). We used the jPRIME analysis to code each taxon for presence or absence of each Group (see Fig. 2). Using Mesquite, we traced the ancestral state with a transition matrix, either setting gain and loss rates to be equal (model MK1), or independent (model MK2), for each of the seven Groups. The MK1 model could not be rejected for any Group, based on the Wilks hypothesis test. We summarized support for ancestral state reconstructions using pie charts showing the proportional likelihoods of alternative states. We considered the state of the node to be resolved (given available information) when one character state contributed over 95% of the proportional likelihood. Lower proportions indicated uncertainty where alternative states could not be ruled out. As an additional test of the statistical support for the orthology of Animal and Fungal sequences within septin groups, we conducted an Approximately Unbiased (AU) test. We constrained the animal septins to be the sister clade to fungal septins and then performed 2000 independent searches with the parameters from the original RAxML analysis to find the maximum likelihood tree given the constraint. We calculated the per-site likelihoods of the data given the most likely constrained and unconstrained tree using RAxML, and used these as input for the AU test with Consel [48].

#### Prediction of interface interacting residues of Group 1 and 2 septins based on crystal structures

To predict septin interface residues, we designed a workflow based on a recent publication on interface residue evolution [49]. First, for each opisthokont taxon, a single Group 1 and a Group 2 septin were chosen. For taxa with multiple paralogs per Group, we arbitrarily chose one protein to represent each Group. Some lineages lacked either a Group 1 or Group 2 septin and we could not include these taxa in the analysis. To increase the size of the dataset, we added *Homo sapiens* Sept2/Sept6 and *Caenorhabditis elegans* unc59/unc61 as they could be assigned to orthologous septin Groups without requiring phylogenetic analysis [50]. This resulted in 17 taxa (34 septins) for further analysis (Additional file 1: Table S2).

Next, we downloaded crystal structures for the Group 1–1 G-interface dimer (2QA5) and Group 2–2 G-interface dimer (3TW4) from PDB [12, 51, 52]. We extracted the Group2–2 NC-interface homodimer, the Group1–2 G-interface heterodimer, and the Group1–2 NC-interface heterodimer from the human septin hexamer (2QAG) using PyMOL [12, 53].

We aligned the appropriate septin pair from each taxon to each of the 5 crystal structures using SALIGN implemented in Modeller [54]. Using Modeller, we produced twenty-five independent homology-folded models of each opisthokont septin dimer, selecting the model with the lowest objective score “molpdf”. To this model we added hydrogen atoms with the program reduce [55] with the flags –build and –FLIP to allow for sidechain rotation based on steric hindrance. We assessed the distance between residues in the reduced dimer structures with the program probe [56] using the default probe diameter of 0.5 Å, with flags -Unformatted to allow parsing of the raw data, -Oneway to ignore chain interactions within a subunit, and -NOCLASHOUT to ignore clashes between the peptide backbones resulting from improper modeling. These settings were selected to only retain interactions from either hydrogen bonding or Van der Waals interactions. We removed duplicate hits from the multiple atoms in a common residue. To correlate interactions with conservation, the locations of the interacting residues were mapped onto *S. cerevisiae* CDC3 (NP_013418) as a reference using an in-house script based on a MAFFT alignment with default settings of all the Group 1 and Group 2 septins in Additional file 1: Table S2.

We assessed the conservation of septin protein sequences by comparing the Jensen-Shannon divergence of residues in each column of an alignment with an entropy-based null model, using the webserver http://compbio.cs.princeton.edu/conservation/ [57] and a window size of one. As input, we used all the septins excluding Group 5 septins, or 103 sequences. We reasoned that since there is no evidence that Group 5 septins bind other core septins [9, 18], they would add noise to the analysis as they likely lack conserved interface residues. To visualize the conservation of the residues that were consistently found interacting in opisthokonts, we used the online server to make WebLogos of the interfaces found in at least 10 of the 17 taxa [58].

## Results

### Septin classification into groups

All sampled opisthokont genomes contained predicted septins, ranging from two in *Sphaeroforma* to 14 in *Phycomyces* (Fig. 2). Previous phylogenetic analysis showed that septins fell into 7 clades designated Group 1A, 1B, 2A, 2B, 3, 4, and 5 (Pan et al., [15]). As a criterion to enable us to compare our Groups to those of Pan et al., we recognized clades based on the previously classified *Aspergillus* ortholog in each (Table 1 and indicated by gene names in bold in Additional file 2: Figure S1–3). Two groups (1B and 2B) lacked *Aspergillus* members, so we recognized these based on *Drosophila* sequences (Table 1, Additional file 2: Figure S1–3, bold gene names). Using these criteria, we could place most opisthokont septins from recently sequenced genomes within the previously recognized clades. The 7 clades accommodated 114/120 sequences in RAxML analysis (Additional file 2: Figure S1), 99/120 in MrBayes analysis (Additional file 2: Figure S2), and all 120 septins with jPRIME (Additional file 2: Figure S3). Use of the partitioned RAxML analysis did not change group assignment compared to the unpartitioned analysis, and was not used further. Septin Groups most commonly contained zero or one gene copy per organism, with some taxa such as *Phycomyces, Basidiobolus,* and *Drosophila* containing duplications in more than one Group (Fig. 2C). Group 1A septins were the most consistently represented across taxa, appearing as single copy genes in 17–20 of 22 taxa (depending on the analysis) and as duplicated genes in one to two taxa. While most fungi had a single copy of a Group 2A septin, *Encephalitozoon* and *Paramicrosporidium* had none, and *Basidiobolus* and *Phycomyces* each had two. Animal lineages, when present, had a single Group 2A septin. *Saccharomyces* had three copies of septins classified as Group 3 using RAxML and jPRIME but these were divergent and difficult to place. In illustrating septin gene copy numbers across septin Groups, the heat map in Fig. 2C shows that three analytical methods, ML, Bayesian and jPRIME largely agreed on the phylogenetic classification of septins.

**Table 1:** Septin nomenclature from selected model organisms. Septins are organized by group based on results of this study, and Pan et al., [15] for *H. sapiens* septins

	1A	1B	2A	2B	3	4	5
*Aspergillus nidulans*	AspD	–	AspB	–	AspA	AspC	AspE
*Saccharomyces cerevisiae*	CDC10	–	CDC3	–	CDC11 Shs1 Spr28	CDC12	–
*Drosophila melanogaster*	–	Sept2/5	–	Sept 1/4/Pnut	–	-Spr3	–
*Homo sapiens*	Sept 3/9/12	Sept 6/8/10/11/14	–	Sept 1/2/4/5/7/13	–	–	–

#### Coiled-coil domains largely follow group designation

The presence or absence of predicted coiled-coil domains was conserved within well-supported septin Groups but sometimes varied when genes were poorly resolved phylogenetically, especially among early-diverging protists. Coiled-coil domains were predicted in almost all members of Groups 1B, 2A, 2B, and 4 (Additional file 2: Figure S1–3). Among Group 1B septins, only *Batrachochytrium* lacked a predicted coiled-coil domain. *Salpingoeca* XP_004994451, placed in Group 2A by the RAxML and MrBayes analyses, seemed to lack the domain, but close inspection showed that the gene prediction was missing the 3′ end where coiled-coil sequences would likely reside if present. Of the Group 3 septins, most (13/19) had a predicted coiled-coil domain. Group 4 septins had predicted coiled-coil domains with the exception of four genes of inconsistent phylogenetic placement (Additional file 2: Figure S1–3). Group 1A lacked coiled-coil domains, again with the exception of poorly supported members such as *Fonticula* XP_009497655 (Additional file 2: Figure S1–3). *Capsaspora* XP_011270180, placed in 1A by jPRIME analysis was unique among our sequences in having a predicted N-terminal coiled-coil domain (Additional file 2: Figure S3). None of the Group 5 septins except *Catenaria* 1512492 had a strongly predicted coiled-coil domain and its domain was positioned in the center of the sequence rather than the end as in other septins. One of the *Basidiobolus* septins also had a weakly predicted coiled-coil domain (Additional file 2: Figure S1–3).

#### Increased taxon sampling captures ancient gene duplications and losses throughout the evolutionary history of septin evolution

To estimate the timing of septin group origins, we performed ancestral state reconstructions. The reconstructions suggested that the ancestor of animals, fungi, and related protists had septin Groups 1A, 4 and possibly 1B, 2 and 5 (Fig. 2A, Additional file 2: Figure S4). Proportional likelihoods helped to distinguish well-supported from uncertain reconstructions. Widely conserved across animals and fungi, Group 1A was reconstructed as ancestral in the Opisthokonts with a high proportional likelihood. Group 4 had a similar high proportional likelihood of ancestral origin in the Opisthokonts. The origin of Group 2 was difficult to place as it was unclear whether 2A and 2B (Pan et al., [15]) formed a monophyletic group (Additional file 2: Figure S1–3). When 2A and 2B were coded separately, the opisthokont ancestor was reconstructed as having neither Group. When 2A and 2B were coded as sister clades, as suggested without statistical support by the jPRIME analysis (Additional file 2: Figure S3), then Group 2 was reconstructed as present in the opisthokont ancestor with 2B evolving from among 2A-like ancestors.

Convergence of septin genes was difficult to rule out and paralogs of different ancestry, possibly under similar selective pressures, may sometimes have come to resemble one another. The monophyly of Group 1B septins was poorly supported (Additional file 2: Figure S3) making convergent origin of the animal vs. fungal Group 1B genes impossible to rule out. Even assuming monophyletic groups were inferred correctly, uncertainty in reconstruction increased as the numbers of ancient inferred gene gains or losses increased. This also was evident in Group 1B, which showed a complicated pattern of gains and losses and was missing from Ichthyosporea but present in other Holozoa; among fungi, it was present in Mucoromycota but missing from Ascomycota and Basidiomycota (Fig. 2C, Additional file 2: Figure S4). The proportional likelihoods correspondingly indicated equivocal support for Group 1B presence or absence throughout early evolution (Additional file 2: Figure S4). Currently available data simply do not resolve where Group 1B septins were gained or lost.

Group 3 was present in most fungi but was missing from *Encephalitozoon*, an early diverging species. Its absence from a basal divergence meant that it was reconstructed with high proportional likelihood only after the divergence of the Microsporidia. Group 5 required more evolutionary transitions to account for its absence from *Gonapodya*, *Conidiobolus* and *Saccharomyces*. The repeated changes made it impossible to resolve whether Group 5 evolved in the ancestor to the Holomycota or deeper in the tree, originating in the ancestor to Opisthokonts followed by loss in the animal lineage (Fig. 2, Additional file 2: Figure S4).

To explain the origin of these Groups, 1–4 gene duplications must have preceded the divergence of fungi from animals. This was consistent with the NOTUNG reconciliation, which indicated the presence of four septins (resulting from three gene duplications) in the opisthokont ancestor prior to the divergence of fungal and animal lineages (Fig. 2B). Among fungi, the ancestor of the walled osmotrophic fungi (*Batrachochytrium* through *Schizophyllum*) gained Group 2A and 3 septins (Fig. 2A). Morphologically simple yeasts (*Saccharomyces* and *Cryptococcus*) had as many septins as the morphologically complex mushroom forming fungus *Schizophyllum*. Among the newly analyzed septins from early diverging animal lineages (*Sphaeroforma* to *Salpingoeca* Fig. 2B and C) and fungal lineages (*Fonticula* to *Gonapodya*, Fig. 2B and C), the representation of septins across the seven Groups is patchy, consistent with repeated gene losses over an immense period of geological time.

As expected, including additional early-diverging lineages not only increased the diversity of septins recovered, but also the challenges involved in phylogenetic resolution (Fig. 2C, Additional file 2: Figure S1–3). All the septins that we analyzed shared a small number of highly conserved sites, without which we could not have recognized and included them. However, outside of the few conserved sites, septins from the early-diverging organisms showed little phylogenetic signal due to minimal amino acid sequence similarity even among sequences that appeared to be orthologs. The absence of recognizable septins from protist relatives of opisthokonts made outgroup rooting of the septin gene tree impossible. We rooted the septin gene tree along a branch present in all inferences (Figs. S1–3) that separated Groups 3 and 5 from Groups 1A, 1B, 2 and 4. This was consistent with midpoint rooting in the RAxML and jPRIME analyses, and this allowed for consistent presentation (Additional file 2: Figure S1–3).

### Highly conserved residues are in GTP binding and monomer interaction surfaces

**Fig. 3:**
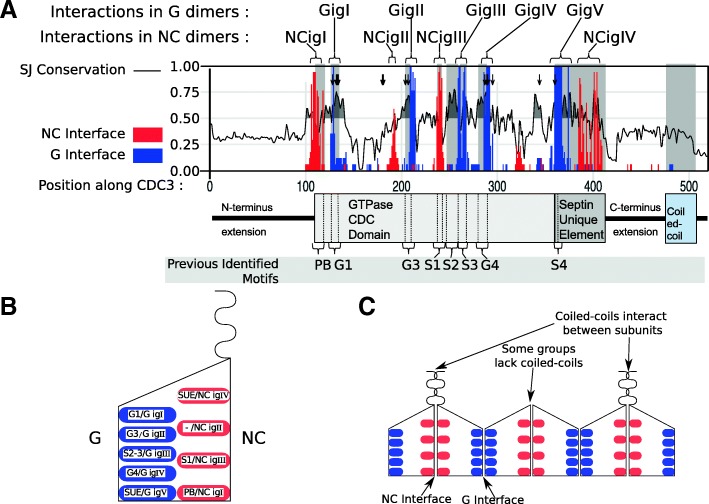
Highly conserved septin residues are involved in GTP-binding and interactions at G- and NC-interfaces. **a**) Conserved residues correspond to predicted interacting residues in interfaces. Solid line represents Shannon-Jensen sequence conservation; shaded curves indicate values above 0.5. Red columns: proportion of taxa where a residue interacts in the NC interface. Blue columns: proportion that interact in the G interface. GTP-binding residues are indicated with black arrows. The generalized diagram of *S. cerevisiae* CDC3 from Pan et al., [15] is shown to scale. **b**) Diagram of a septin monomer showing the organization of interface residues at the NC and G interfaces. The curved line at the top represents a coiled-coil. **c**) Model showing how monomers interact to form heterooligomers. The interacting group (ig) residues, colored as in **b**), indicate predicted residue interactions between septin partners. Not to scale

Previous analysis of opisthokont septins revealed three highly conserved motifs involved in GTP binding (G1, G3 and G4) along with five motifs of unknown function (S1, S2, S3, S4 and SUE) [15]. To determine if these conserved residues were also present in septins from recently sequenced early-diverging opisthokonts, we analyzed the Shannon-Jensen conservation of the phylogenetic dataset (Fig. 3) [57]. As expected, all three GTP binding motifs were highly conserved. Like the G boxes, the S2 motif was highly conserved. The S1, S3, S4 and the SUE motifs were conserved but to a somewhat lesser extent. When aligned to the *Saccharomyces cerevisiae* CDC3 reference septin, 147/520 residues (28%) had a Shannon-Jensen conservation score greater than 0.5 and all residues with scores above 0.5 were within the central region of the protein.

To find the positions of the highly-conserved residues within septin filaments, we modeled 14 orthologous opisthokont septin pairs based on five known structures of *Homo sapiens* dimers, focusing on interface regions between monomers (Fig. 3, Additional file 2: Figure S5) [12, 52]. The alternative crystal structures of the G and NC interfaces each have different regions that were not resolved, and thus any one crystal structure was insufficient to reveal all interacting sites (reviewed in Valadares, Pereira, Araujo, & Garratt, 2017, see Additional file 2: Figure S5). To identify residues consistently involved in septin-septin interactions, we used a criterion of interaction in the modelled dimers of at least 10 of the 17 taxa in at least one of the crystal structures. The conservation of interface residues is shown in Fig. 3 and Additional file 2: Figure S5. As an example, G dimers modelled on the human Sept7 dimer (3TW4 from [52], Additional file 2: Figure S5) show interactions near the G3 box not apparent in other G interface dimers, but lack resolution near the S4 region inside the SUE found in other dimers. We found 29 interacting residues across the G interface and 20 across the NC interface. An alignment of interaction residues in representative septins is shown in Additional file 2: Figure S6. All previously defined conserved septin motifs included interface residues, often in close proximity to additional interface residues. In the G interface, 22 of the 29 residues were in previously identified regions/motifs, and 14 of the 20 residues in the NC interface were in previously identified regions/motifs.

#### Conservation of interface residues varies among septin groups

**Fig. 4:**
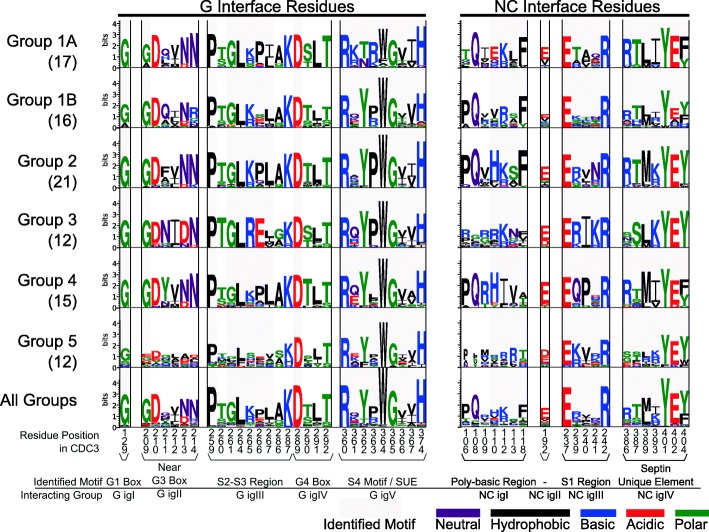
Patterns of diversity and conservation across septin groups. WebLogo showing patterns of amino acid conservation and diversity across aligned motifs and domains of the septin Groups. Interacting residues are split into the G and the NC interfaces, and subdivisions into interacting groups are shown below. Residues are numbered according to the COBALT aligned position with *S. cerevisiae* CDC3. In parentheses below each Group number is the number of sequences used to build the WebLogo

Septins from the five different groups interact with each other in a reproducible manner, and conservation of the interface residues may explain these interactions. To investigate conservation of interface residues we used WebLogo to analyze the 29 residues of the G interface and the 20 residues of the NC interface within each septin group (Fig. 4). To allow comparison, we numbered residues relative to the reference *Saccharomyces* Group 2 septin CDC3. Overall, the Group 5 septins showed lower conservation of interface residues, though D289, R360, W364, and H374, which were highly conserved across all septin groups were also conserved in Group 5. The other septin Groups showed higher overall interface residue conservation. Position 129 in the G1 Box was strongly conserved with glycine for all Groups (Fig. 4). Near the G3 box, G209 and D210 were highly conserved, and positions 211, 213, and 214 showed conserved differences of amino acids of differing classes across Groups. In the S2-S3 region, the only difference of note was an acidic glutamate in Group 3 at site 266 that replaced the proline in Groups 1A/2/4. The G4 box did not have any interface residues that differed notably among Groups. The G interface residues in the SUE were all in the same residue class, except for position 361, which was variable both among and within Groups (Fig. 4).

The 20 residues of the NC interface showed similar patterns (Fig. 4), with Group 5 septins having the lowest overall conservation. The PB region had many differences among Groups. Notably Group 3 and Group 5 had strikingly low sequence conservation across the whole PB region (NC interacting group I), the region corresponding to the α0 helix that is unique to septins. The distance between the start of the N-terminus and the PB region is short in both Group 3 and 5 proteins (Additional file 2: Figure S5) compared with other septins suggesting that the α0 helix might be truncated or otherwise altered in these groups.

Position 192 was found as an interacting residue, usually of glutamate, but it was neither highly conserved nor close to a previously defined conserved region. In the S1 region, the glutamate at 237 and the arginine at 242 were highly conserved in all Groups, but 239 was variable among Groups. Positions 240 and 241 were variable both among and within Groups. The SUE contained conserved residues such as tyrosine/glutamate at 401–2. Position 386 was arginine in some Groups, but was not conserved in other Groups. Position 393 was a mix of basic lysine and arginine in Groups 2/3/5 and hydrophobic isoleucine in Groups 1A/1B/4. Position 404 varied between tyrosine and phenylalanine, both bulky aromatic amino residues.

## Discussion

Septin groups are more ancient than previously realized, with early diverging lineages having a broader representation than previously realized. Pan et al. [15] had been unable to differentiate whether Group 5 arose early and was lost in Ascomycete yeasts, or was gained relatively recently in fungal evolution. Improved sampling of septins from early-diverging lineages brought clarity, showing that Group 5 was ancestral in the clade of fungi from Chytridiomycota to Basidiomycota (see Fig. 2), and may have arisen even earlier. Our analysis suggested that in addition to Groups 1A and 4, septin Groups 1B and 2 may also have been present in the opisthokont ancestor. Cao et al. (2007) concluded that septin Groups diversified before vertebrate and invertebrate animals diverged. Our results show evidence of Holozoan septin diversification prior even to the origin of multicellularity in animals. Like Pan et al. [15], we found strong evidence for orthologous Group 1A septins among animals and fungi. We tracked Group 1A septin sequences in newly available genomes, including diverse early diverging animal genomes from *Trichoplax*, a placozoan multicellular animal, and *Monosiga*, a collar flagellate. Poor resolution in the phylogeny of early diverging septins made precise localization of some septin duplication events impossible. However, imperfect though the phylogenetic resolution sometimes was, the improved taxon sampling in our analyses contributed to revealing underlying complexity in patterns of gene evolution.

Our analysis suggested that the Opisthokont common ancestor contained both a septin with and a septin without a coiled-coil domain, arising from an ancestral gene duplication. Septins have been detected in green algae, brown algae and ciliates, indicating that they may have arisen very early in the eukaryotic tree of life [59]. However, the non-opisthokont septins lack a coiled-coil domain [59]. On the other hand, each opisthokont species in our analysis except *Paramicrosporidium* had one or more septins with and one or more without the coiled-coil. Monomers with coiled-coil domains in *Saccharomyces,* Group 4 CDC12, and Group 2B CDC3 are essential for polymerization of septins into normal, stable octamers as well as for their further organization into filaments [60, 61]. Deletion of the coiled-coil domain produced aberrant morphology in the ascomycete yeasts *S. cerevisiae* and *Ashbya gossypii* [61, 62]. The role of septin monomers without coiled-coil domains is less clear, but in *Saccharomyces*, a pair of Group 1A monomers (Cdc10), and in *Homo* hexamers a pair of Group 2A monomers (Sept2) form the central doublet in octameric septin rods [12, 61]. In *Aspergillus* the ortholog AspD is non-essential even though it is inferred to form the central dimer of the *Aspergillus* octamer [63]. The conserved absence of a coiled-coil domain from almost all Group 1A members and the conserved presence of the domain through Groups 1B, 2, and 4 has apparently persisted over hundreds of millions of years of evolution, consistent with selection for different but important functions.

Our analysis of the residues involved in septin-septin interactions suggested roles for previously discovered S1–4 conserved domains [15]. By using homology folding across the breadth of evolutionary diversity in opisthokonts, we showed that the S domains are all predicted to be involved in interface interactions. We also recovered evidence of interface interactions in the alpha helix found in the polybasic region, consistent with earlier work from Agelis & Spiliotis [5]. While almost all of the putative interface interactions that we detected were in regions and motifs previously identified as highly conserved, the glutamic acid identified as an NC interface residue at position 192 had not previously been noted. The general correlation of interface residues with conserved gene sequences is consistent with evolutionary expectations. The requirement for interactions between residues constrains the sequence evolution in these regions, as both partners would require compensatory mutations for the heterooligomer to form properly. Our finding that Group 5 septins show little sequence conservation in interface regions is consistent with evidence that they do not form part of the core septin oligomer (Hernádez-Rodríguez et al. [18]).

Septin-septin interactions are thought to be governed largely by the residues in the G- and NC-interfaces, possibly with input from coiled-coil domain interactions. The G interface was generally more conserved than the NC interface, which is not surprising since its role in binding GTP further limits changes in sequence. Exemplifying this importance is the highly-conserved tryptophan in the G interface (position 364 in Fig. 4), which only showed divergence in the Group 1A septins. Mutagenizing this bulky aromatic to a much smaller alanine eliminated dimerization of Group 3 septins in yeast [64]. The differences among groups in residues in G- and NC-interfaces could explain the preferential affinity of the individual Groups. Some septin Groups differed at one or two interface residues. Asparagine was, for example, conserved at site 211 within Group 3 but tyrosine was conserved at the same site in Group 4. The substitution of an amino acid with different chemical properties potentially affects interface binding.

## Conclusion

By including a diverse set of early-diverging opisthokont lineages, we recovered more diverse sequences than were analyzed previously, allowing us to show that septin duplications were ancient with up to four septin paralogs in the opisthokont ancestor. With alignments of these diverse sequences and homology folding, we found that interface residue conservation overlapped with evolutionarily conserved residues, indicating the tight relationship between septin partners over time. Septins with coiled-coil domains were ancient in the opisthokonts, suggesting that not only septin heterooligomers but also higher order filaments were part of the ancestral cellular tool kit of both animals and fungi.

## Additional files

Additional file 1: **Table S1.** Sources for proteomes used in this study. **Table S2.** Sequences used in homology modelling. Joint Genome Institute protein IDs are given for *B. meritosporus* and *C. coronatus*. For other taxa, protein codes are GenBank accession numbers. (ZIP 193 kb)

Additional file 2: **Figure S1.** Maximum likelihood phylogenetic analysis with RAxML software. Node values represent bootstrap support. Protein names are given for septins supported by experimental evidence. *Aspergillus* and *Drosophila* sequences used to recognize septin groups are in bold. Coiled-coil domain predictions, black representing *p* < 0.05 and grey *p* < 0.10, found to the right of names. Domain predictions for proteins longer than 600 residues have been shortened with diagonal lines. **Figure S2.** Bayesian phylogenetic analysis. Node values represent posterior probabilities. Protein names are shown for those septins with experimental evidence. *Aspergillus* and *Drosophila* sequences used to recognize septin groups are in bold. Coiled-coil domain prediction, black representing *p* < 0.05 and grey *p* < 0.10, shown to the right of names. Coiled-coil predictions for proteins longer than 600 residues have been shortened with diagonal lines. **Figure S3.** Bayesian phylogeny with jPRIME software. Topology represents maximum clade credibility tree; node values represent bootstrap support. Protein names are shown for those septins with experimental evidence. *Aspergillus* and *Drosophila* sequences used to recognize septin groups are in bold. Coiled-coil domain prediction, black representing *p* < 0.05 and grey *p* < 0.10 to the right of septin names. Coiled-coil predictions for proteins longer than 600 residues have been shortened with diagonal lines. **Figure S4.** Ancestral state reconstructions for presence of septin groups inferred using Mesquite with the MK1 symmetrical model. Shading of pie charts at nodes represent proportional likelihood of a node containing a member of that septin group. Statistical test showing that MK1 could not be rejected appears below state reconstructions. This test supports assuming a single rate of change for gains and losses. **Figure S5.** Interacting residues. A) Interacting residues found based on modelling the 5 individual crystal structures. Red or blue shading indicates the proportion of taxa for which a given residue interacts in the NC or G interface, respectively. Note that no single crystal structure alone can be used to assess all interface regions due to low resolution portions in each crystal. Crystal structures used were as follows: 3TW4 (Human Sept7) provided the Group 1–1 Homodimer interface. 2QA5 (Human Sept 2/6/7 hexamer) provided the Group 2–2 homodimer G and NC interface, and the Group 2–1 NC interface. 2QAG (Human Sept2 dimer) provided the Group 2–2 homodimer G interface. B) Interface conservation as in Fig. 3A, with the solid line representing Shannon-Jensen sequence conservation; shading indicates values above 0.5. GTP-biding residues are indicated with black arrows, with sequential residues having overlapping arrows. The red and blue columns indicate the highest value for a position from the individual crystal structures in A). **Figure S6.** COBALT alignment of representative septins from the 7 groups, showing location of conserved regions and interface regions in a representative septin from each of the seven groups. Green cylinders represent position of alpha helices, and pink arrow indicate beta sheets. Consistently interacting residues are indicated by blue for G interface, and red for NC interface. Motifs identified by Pan et al., [15] are outlined in black boxes. (ZIP 2531 kb)

## Abbreviations

CDC3: Cell Division Cycle 3 - Group 2 septin from the yeast *Saccharomyces cerevisiae*, often used as a reference septin
CTE: C-Terminal Extension: The carboxy terminal end of a septin protein that is highly variable across septins groups
NTE: N-Terminal Extension: The amino terminal end of a septin protein that is highly variable across septin groups
PB: Polybasic Domain: Region of a septin protein found amino-terminal to the GTP binding domain, largely composed of basic residues. Also referred to as the Phosphoinositol Binding domain due to its requirement for septins to bind phosphoinositol
