# Supplementary material for: Diversity of opisthokont septin proteins reveals structural constraints and conserved motifs
Source: BMC Evol Biol. 2019 Jan 7;19:4. doi: 10.1186/s12862-018-1297-8 (PMC6323724; doi:10.1186/s12862-018-1297-8)
Supplement: Supplementary file 1 — Table S1. Sources for proteomes used in this study. Table S2. Sequences used in homology modelling. Joint Genome Institute protein IDs are given for B. meritosporus and C. coronatus. For other taxa, protein codes are GenBank accession numbers. (ZIP 193 kb) [file 12862_2018_1297_MOESM1_ESM.zip › Table S1.docx]

Table S1: Sources for proteomes used in this study.

| Organism | Genome Version Info | Source |
| --- | --- | --- |
| *Allomyces macrogynus* | GCA_000151295.1 / A_macrogynus_V3 | NCBI |
| *Aspergillus nidulans* | GCF_000149205.1 / ASM14920V1 | NCBI |
| *Basidiobolus meristosporus* | CBS 931.73 v1.0 | JGI |
| *Batrachochytrium dendrobatidis* | GCA_000203795.1 / v1.0 | NCBI |
| *Catenaria anguillulae* | PL171 v2.0 | JGI |
| *Capsaspora owczarzaki* | GCA_000151315.2 / C_owczarzaki_V2 | NCBI |
| *Conidiobolus coronatus* | GCA_001566745.1 | NCBI |
| *Creolimax fragrantissima* | Accessed Sep 26, 2016  DOI: 10.6084/m9.figshare.1403592 | FigShare |
| *Cryptococcus neoformans* | GCA_000149245.3 / CNA3 | NCBI |
| *Drosophila melanogaster* | GCA_000001215.4 / Release 6 plus ISO1 MT | NCBI |
| *Encephalitozoon cuniculi* | GCA_000091225.1 / ASM9122V1 | NCBI |
| *Fonticula alibcans* | GCA_000388065.2 / Font_alba_ATCC_28817_V2 | NCBI |
| *Gonapodya prolifera* | GCA_001574975.1 / Ganpr1 | NCBI |
| *Monosiga brevicollis* | GCA_000002865.1 / V1.0 | NCBI |
| *Paramicrosporidium saccamaboe* | draft | Dr. Alicia Quandt |
| *Phycomyces blakesleeanus* | GCA_001638985.2 / Phybl2 | NCBI |
| *Rhizophagus irregularis* | GCA_001593125.1 / ASM159312v1 | NCBI |
| *Saccharomyces cerevisiae* | GCA_000146045.2 / R64 | NCBI |
| *Salpingoeca rosetta* | GCA_000188695.1 / Proterospongia_sp_ATCC50818 | NCBI |
| *Schizophyllum commune* | GCA_000143185.1 / V1.0 | NCBI |
| *Sphaeroforma arctica* | GCA_001186125.1 / Spha_artica_JP610_V1 | NCBI |
| *Trichoplax adhaerens* | GCA_000150275.1 / v1.0 | NCBI |
