# Supplementary material for: Diversity of opisthokont septin proteins reveals structural constraints and conserved motifs
Source: BMC Evol Biol. 2019 Jan 7;19:4. doi: 10.1186/s12862-018-1297-8 (PMC6323724; doi:10.1186/s12862-018-1297-8)
Supplement: Supplementary file 1 — Table S1. Sources for proteomes used in this study. Table S2. Sequences used in homology modelling. Joint Genome Institute protein IDs are given for B. meritosporus and C. coronatus. For other taxa, protein codes are GenBank accession numbers. (ZIP 193 kb) [file 12862_2018_1297_MOESM1_ESM.zip › Table S2.no_line_numbers.docx]

Table S2: Sequences used in homology modelling. Protein codes represent GenBank accession numbers except for *B. meritosporus* and *C. coronatus* which are not listed on GenBank and instead use Joint Genome Institute protein IDs.

| Taxonomic  Group | Taxa | Group 1 Septin | Group 2 Septin |
| --- | --- | --- | --- |
| Dikarya | *Aspergillus nidulans* | XP_664292.1 | XP_658998.1 |
|  | *Cryptococcus neoformans* | XP_012050631.1 | XP_012049501.1 |
|  | *Saccharomyces cerevisae* | NP_013418.2 | NP_009928.1 |
|  | *Schizophyllum commune* | XP_003037204.1 | XP_003035685.1 |
| Early-diverging Fungi | *Allomyces macrogynus* | KNE60169.1 | KNE63007.1 |
|  | *Basidiobolus meritosporus* | 313179 | 315462 |
|  | *Batrachochtytrium dendrobatidis* | XP_006675869.1 | XP_006678596.1 |
|  | *Catenaria anguillulae* | 1173908 | 1512492 |
|  | *Conidiobolus coronatus* | KXN68162.1 | KXN74403.1 |
|  | *Gonapodya prolifera* | KXS17957.1 | KXS10614.1 |
|  | *Phycomyces blakesleeanus* | OAD67191.1 | OAD79262.1 |
| Animals | *Drosophila melanogaster* | NP_523430.1 | NP_524417.1 |
|  | *Caenorhabditis elegans* | NP_001041198 | NP_493388 |
|  | *Homo sapiens* | XP_011529619 | XP_016859678 |
|  | *Trichoplax adherans* | XP_002112086.1 | XP_002112436.1 |
| Early-diverging Metazoa | *Monosiga brevicola* | XP_001742566.1 | XP_001745238.1 |
|  | *Capsaspora owczarski* | XP_004349897.2 | XP_004346425.2 |
