# Supplementary figures and images for: Diversity of opisthokont septin proteins reveals structural constraints and conserved motifs
Source: BMC Evol Biol. 2019 Jan 7;19:4. doi: 10.1186/s12862-018-1297-8 (PMC6323724; doi:10.1186/s12862-018-1297-8)

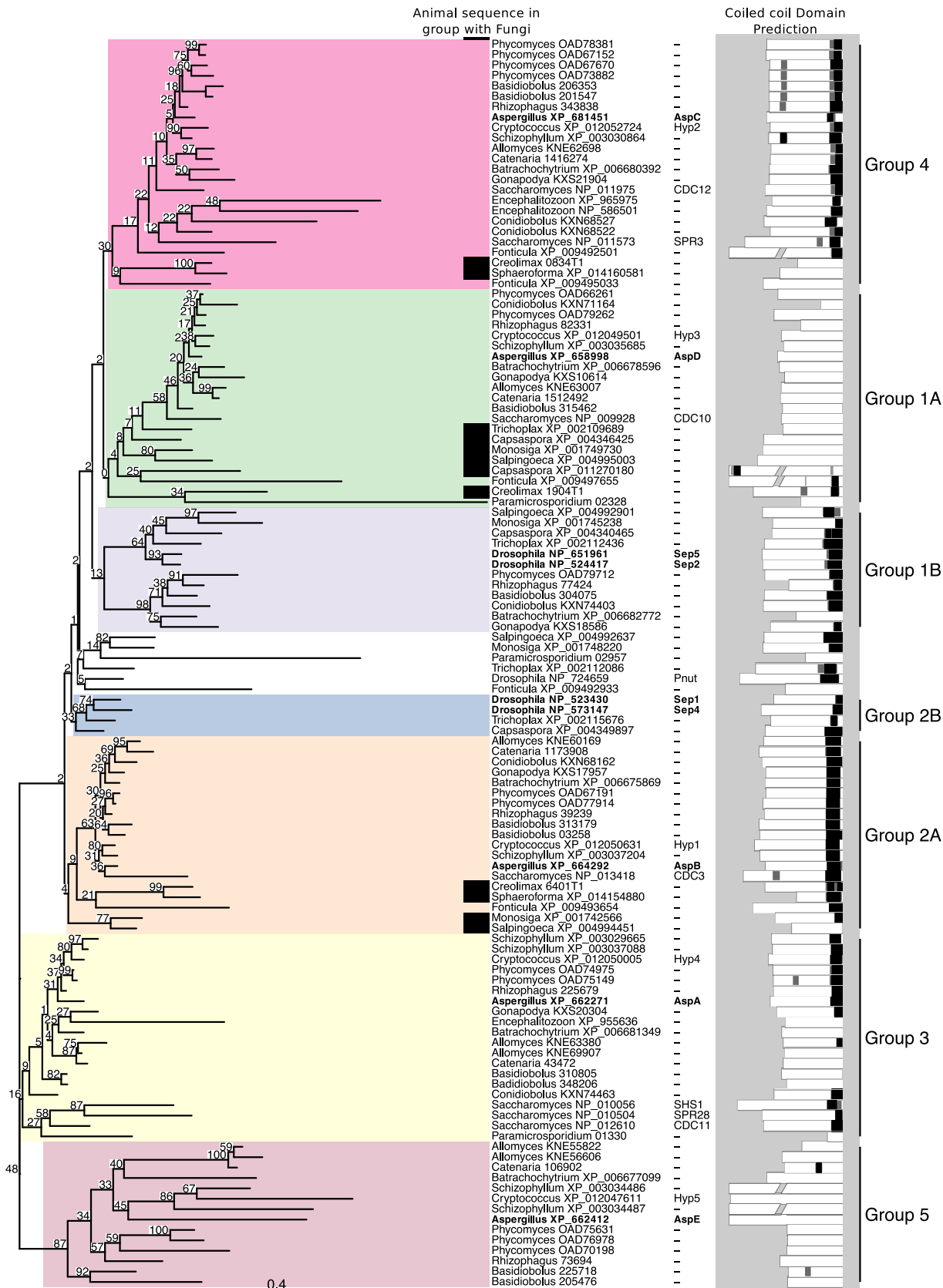

Supplement: Supplementary file 2 — Figure S1. Maximum likelihood phylogenetic analysis with RAxML software. Node values represent bootstrap support. Protein names are given for septins supported by experimental evidence. Aspergillus and Drosophila sequences used to recognize septin groups are in bold. Coiled-coil domain predictions, black representing p < 0.05 and grey p < 0.10, found to the right of names. Domain predictions for proteins longer than 600 residues have been shortened with diagonal lines. Figure S2. Bayesian phylogenetic analysis. Node values represent posterior probabilities. Protein names are shown for those septins with experimental evidence. Aspergillus and Drosophila sequences used to recognize septin groups are in bold. Coiled-coil domain prediction, black representing p < 0.05 and grey p < 0.10, shown to the right of names. Coiled-coil predictions for proteins longer than 600 residues have been shortened with diagonal lines. Figure S3. Bayesian phylogeny with jPRIME software. Topology represents maximum clade credibility tree; node values represent bootstrap support. Protein names are shown for those septins with experimental evidence. Aspergillus and Drosophila sequences used to recognize septin groups are in bold. Coiled-coil domain prediction, black representing p < 0.05 and grey p < 0.10 to the right of septin names. Coiled-coil predictions for proteins longer than 600 residues have been shortened with diagonal lines. Figure S4. Ancestral state reconstructions for presence of septin groups inferred using Mesquite with the MK1 symmetrical model. Shading of pie charts at nodes represent proportional likelihood of a node containing a member of that septin group. Statistical test showing that MK1 could not be rejected appears below state reconstructions. This test supports assuming a single rate of change for gains and losses. Figure S5. Interacting residues. A) Interacting residues found based on modelling the 5 individual crystal structures. Red or blue shading indicates the p [file 12862_2018_1297_MOESM2_ESM.zip › FigS1.RAxML.phylogeny.flat.pdf]

# Presence of Animal with Fungi in Group

# Coiled coil Domain Prediction

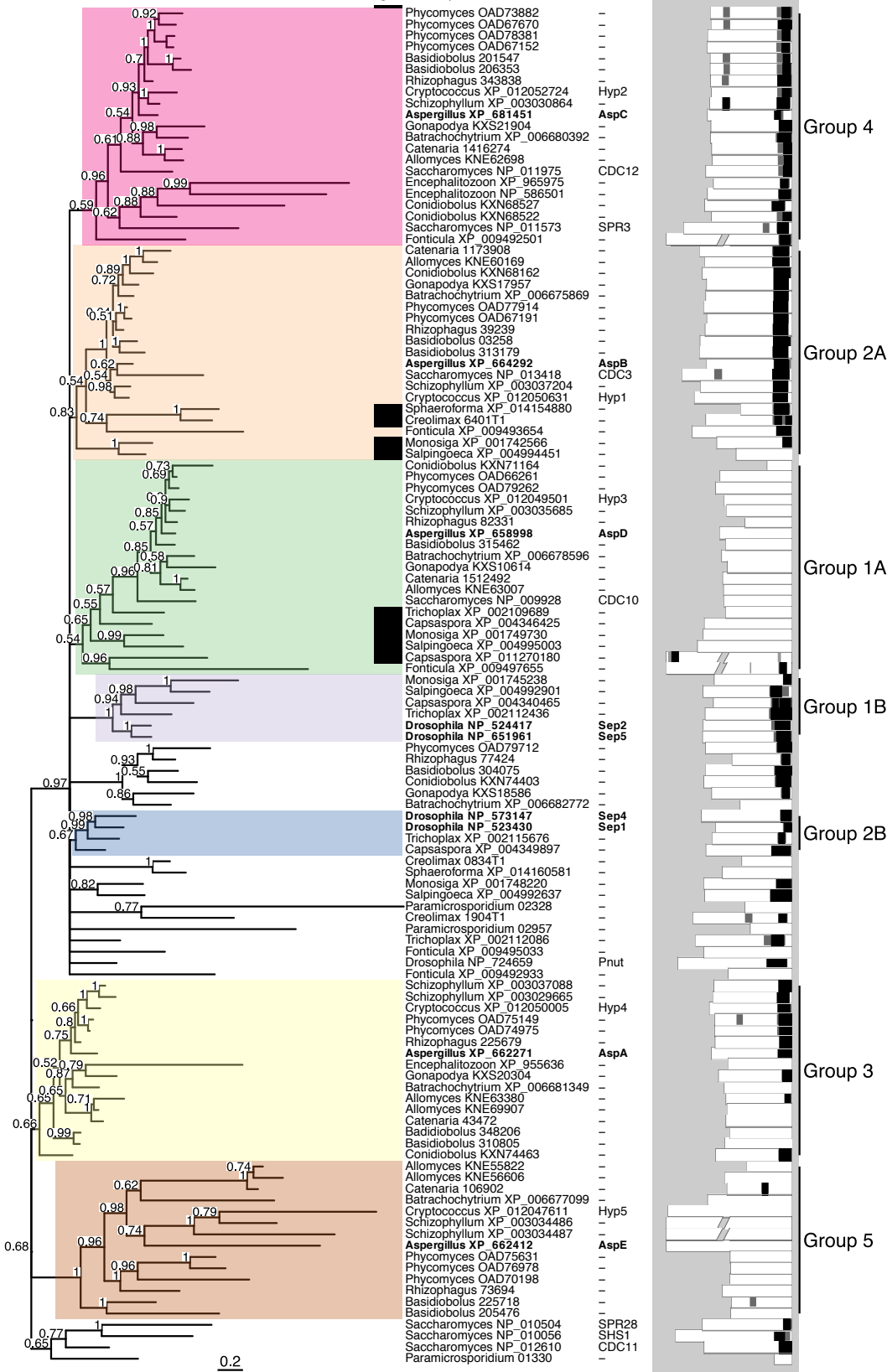

Supplement: Supplementary file 2 — Figure S1. Maximum likelihood phylogenetic analysis with RAxML software. Node values represent bootstrap support. Protein names are given for septins supported by experimental evidence. Aspergillus and Drosophila sequences used to recognize septin groups are in bold. Coiled-coil domain predictions, black representing p < 0.05 and grey p < 0.10, found to the right of names. Domain predictions for proteins longer than 600 residues have been shortened with diagonal lines. Figure S2. Bayesian phylogenetic analysis. Node values represent posterior probabilities. Protein names are shown for those septins with experimental evidence. Aspergillus and Drosophila sequences used to recognize septin groups are in bold. Coiled-coil domain prediction, black representing p < 0.05 and grey p < 0.10, shown to the right of names. Coiled-coil predictions for proteins longer than 600 residues have been shortened with diagonal lines. Figure S3. Bayesian phylogeny with jPRIME software. Topology represents maximum clade credibility tree; node values represent bootstrap support. Protein names are shown for those septins with experimental evidence. Aspergillus and Drosophila sequences used to recognize septin groups are in bold. Coiled-coil domain prediction, black representing p < 0.05 and grey p < 0.10 to the right of septin names. Coiled-coil predictions for proteins longer than 600 residues have been shortened with diagonal lines. Figure S4. Ancestral state reconstructions for presence of septin groups inferred using Mesquite with the MK1 symmetrical model. Shading of pie charts at nodes represent proportional likelihood of a node containing a member of that septin group. Statistical test showing that MK1 could not be rejected appears below state reconstructions. This test supports assuming a single rate of change for gains and losses. Figure S5. Interacting residues. A) Interacting residues found based on modelling the 5 individual crystal structures. Red or blue shading indicates the p [file 12862_2018_1297_MOESM2_ESM.zip › FigS2.MrBayes.phylogeny.flat.pdf]

# Animal taxa in group with Fungi

# Coiled coil Domain Prediction

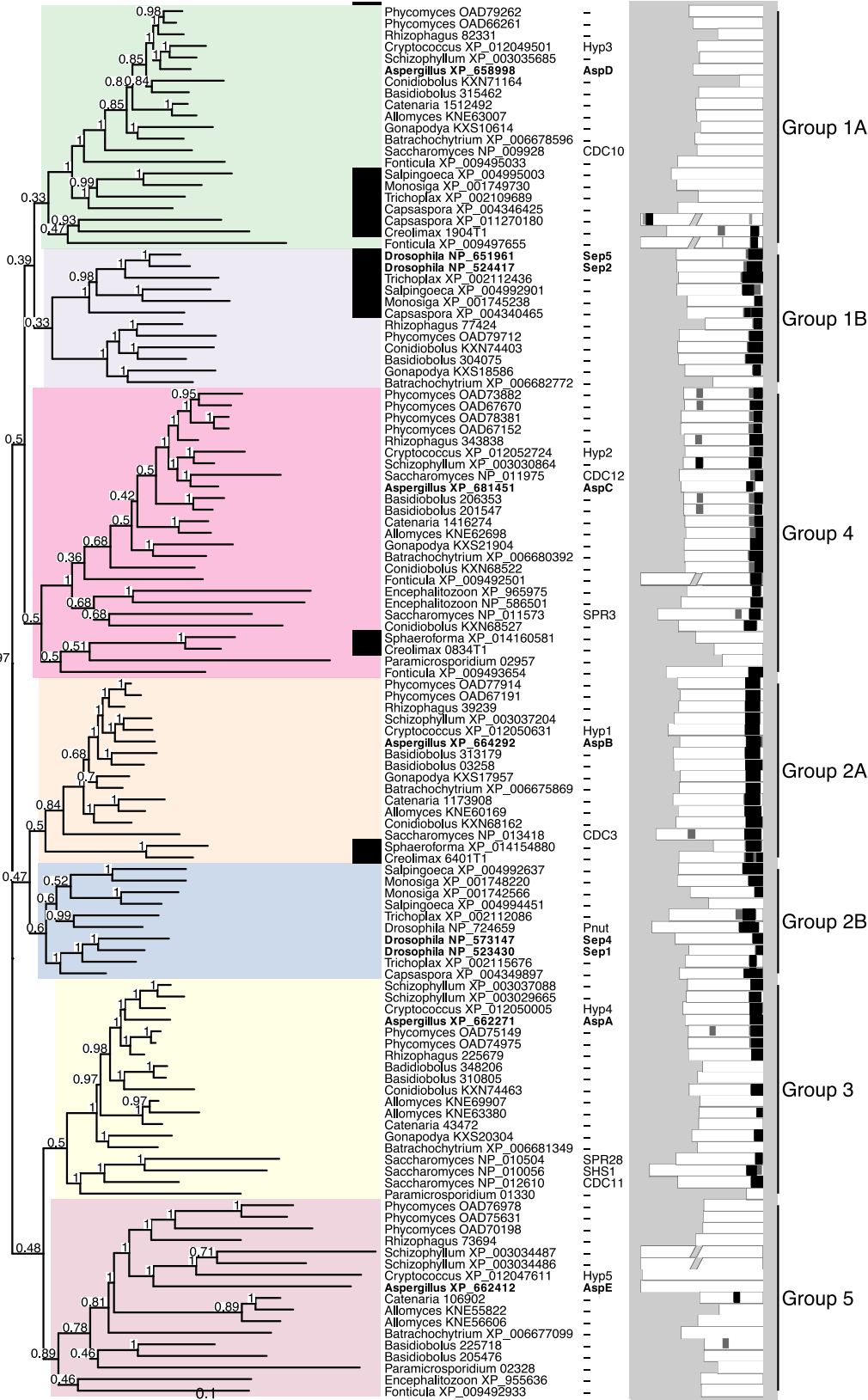

Supplement: Supplementary file 2 — Figure S1. Maximum likelihood phylogenetic analysis with RAxML software. Node values represent bootstrap support. Protein names are given for septins supported by experimental evidence. Aspergillus and Drosophila sequences used to recognize septin groups are in bold. Coiled-coil domain predictions, black representing p < 0.05 and grey p < 0.10, found to the right of names. Domain predictions for proteins longer than 600 residues have been shortened with diagonal lines. Figure S2. Bayesian phylogenetic analysis. Node values represent posterior probabilities. Protein names are shown for those septins with experimental evidence. Aspergillus and Drosophila sequences used to recognize septin groups are in bold. Coiled-coil domain prediction, black representing p < 0.05 and grey p < 0.10, shown to the right of names. Coiled-coil predictions for proteins longer than 600 residues have been shortened with diagonal lines. Figure S3. Bayesian phylogeny with jPRIME software. Topology represents maximum clade credibility tree; node values represent bootstrap support. Protein names are shown for those septins with experimental evidence. Aspergillus and Drosophila sequences used to recognize septin groups are in bold. Coiled-coil domain prediction, black representing p < 0.05 and grey p < 0.10 to the right of septin names. Coiled-coil predictions for proteins longer than 600 residues have been shortened with diagonal lines. Figure S4. Ancestral state reconstructions for presence of septin groups inferred using Mesquite with the MK1 symmetrical model. Shading of pie charts at nodes represent proportional likelihood of a node containing a member of that septin group. Statistical test showing that MK1 could not be rejected appears below state reconstructions. This test supports assuming a single rate of change for gains and losses. Figure S5. Interacting residues. A) Interacting residues found based on modelling the 5 individual crystal structures. Red or blue shading indicates the p [file 12862_2018_1297_MOESM2_ESM.zip › FigS3.jPRIME.phylogeny.flat.pdf]

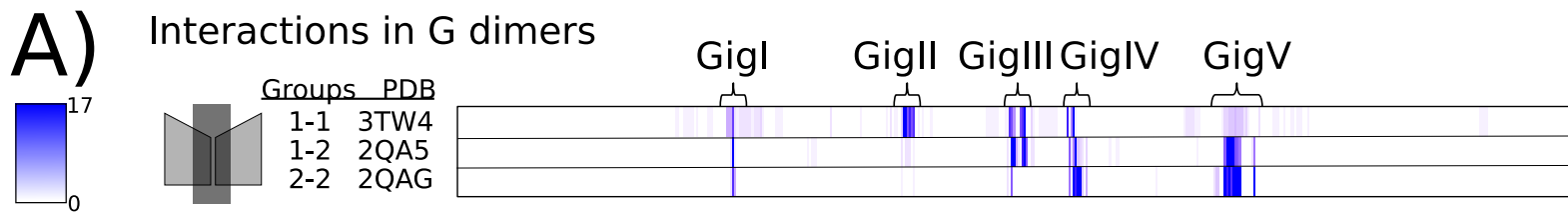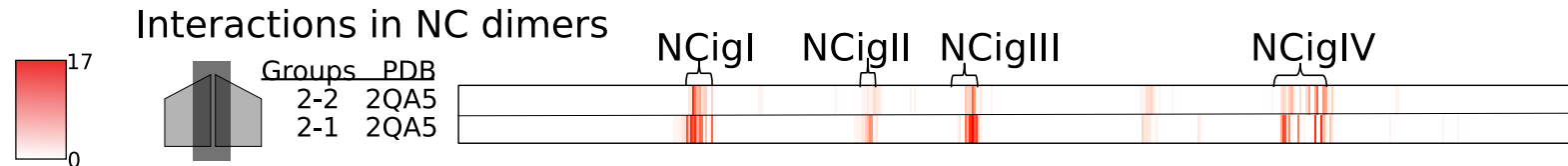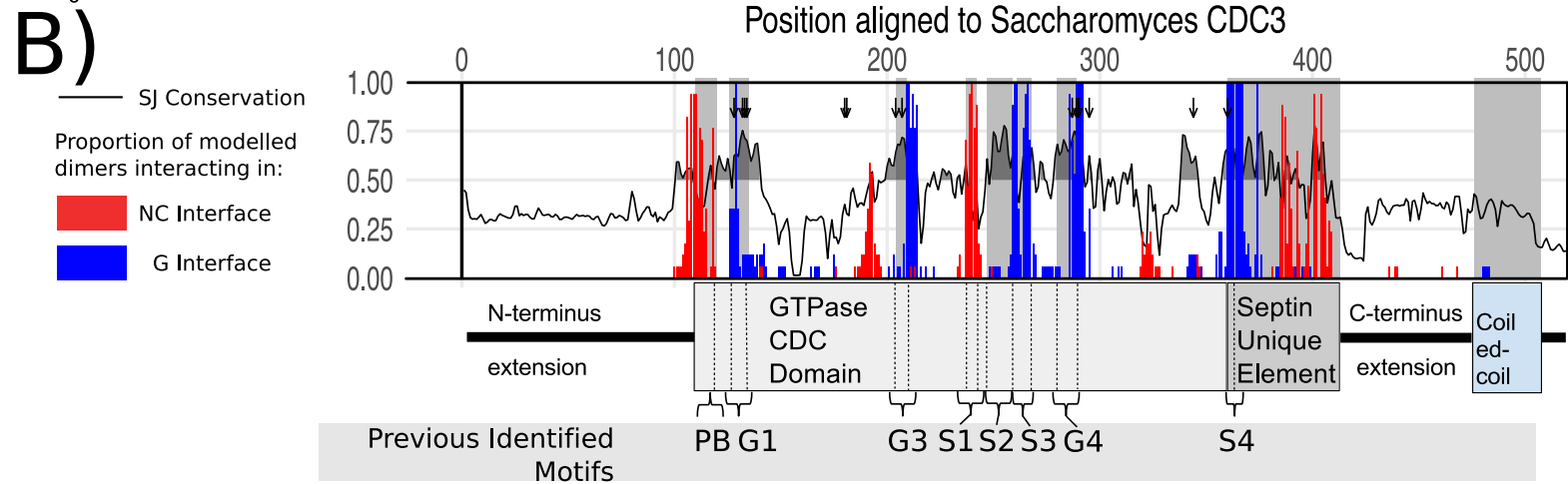

Supplement: Supplementary file 2 — Figure S1. Maximum likelihood phylogenetic analysis with RAxML software. Node values represent bootstrap support. Protein names are given for septins supported by experimental evidence. Aspergillus and Drosophila sequences used to recognize septin groups are in bold. Coiled-coil domain predictions, black representing p < 0.05 and grey p < 0.10, found to the right of names. Domain predictions for proteins longer than 600 residues have been shortened with diagonal lines. Figure S2. Bayesian phylogenetic analysis. Node values represent posterior probabilities. Protein names are shown for those septins with experimental evidence. Aspergillus and Drosophila sequences used to recognize septin groups are in bold. Coiled-coil domain prediction, black representing p < 0.05 and grey p < 0.10, shown to the right of names. Coiled-coil predictions for proteins longer than 600 residues have been shortened with diagonal lines. Figure S3. Bayesian phylogeny with jPRIME software. Topology represents maximum clade credibility tree; node values represent bootstrap support. Protein names are shown for those septins with experimental evidence. Aspergillus and Drosophila sequences used to recognize septin groups are in bold. Coiled-coil domain prediction, black representing p < 0.05 and grey p < 0.10 to the right of septin names. Coiled-coil predictions for proteins longer than 600 residues have been shortened with diagonal lines. Figure S4. Ancestral state reconstructions for presence of septin groups inferred using Mesquite with the MK1 symmetrical model. Shading of pie charts at nodes represent proportional likelihood of a node containing a member of that septin group. Statistical test showing that MK1 could not be rejected appears below state reconstructions. This test supports assuming a single rate of change for gains and losses. Figure S5. Interacting residues. A) Interacting residues found based on modelling the 5 individual crystal structures. Red or blue shading indicates the p [file 12862_2018_1297_MOESM2_ESM.zip › FigS5.Feb19.conservation.pdf]

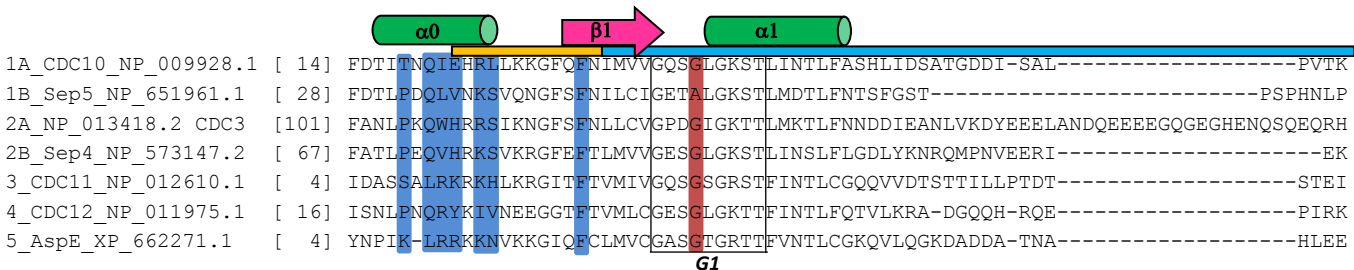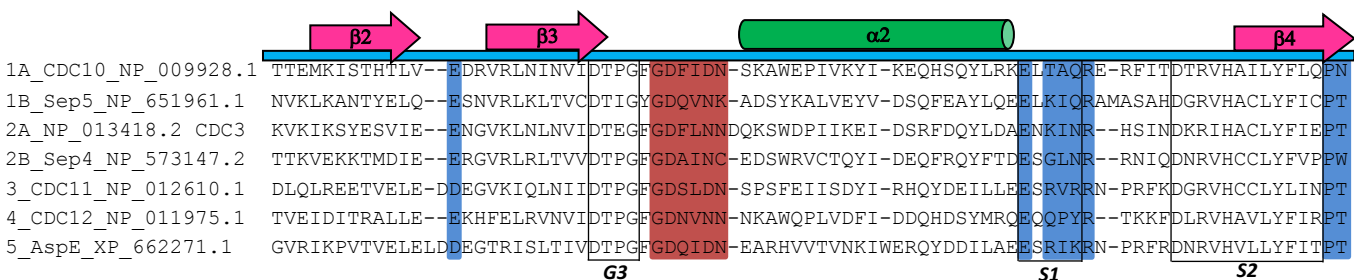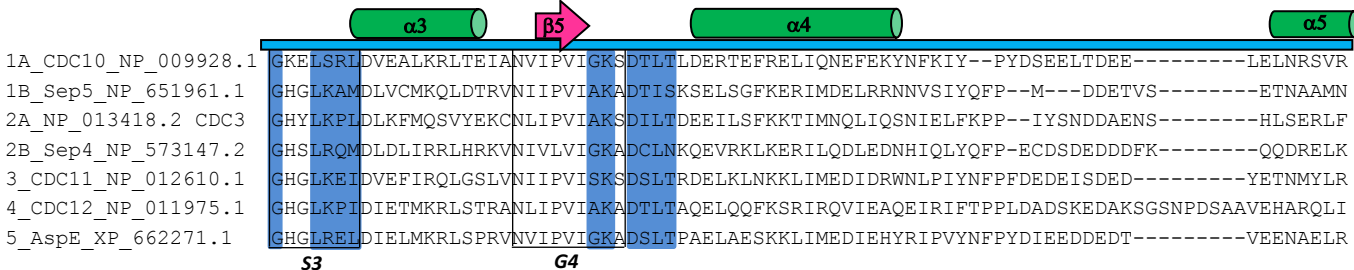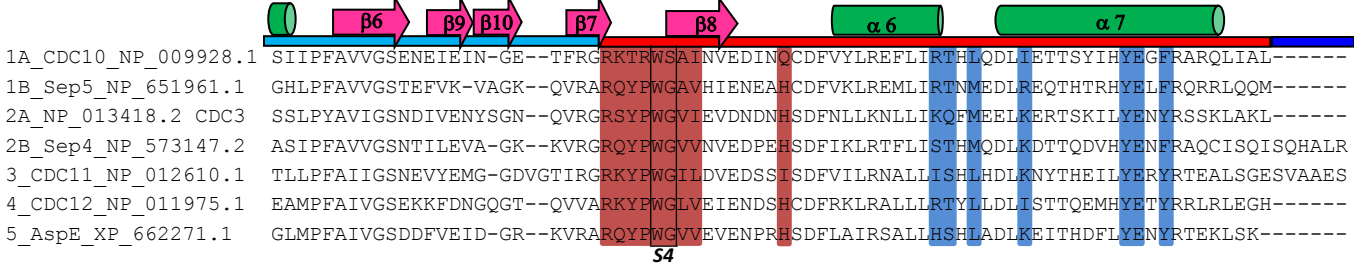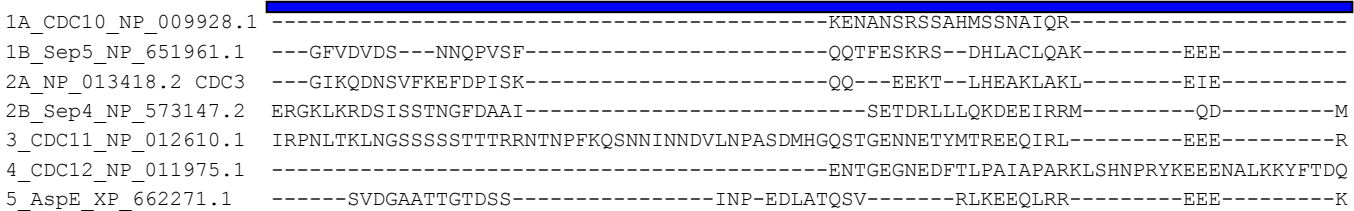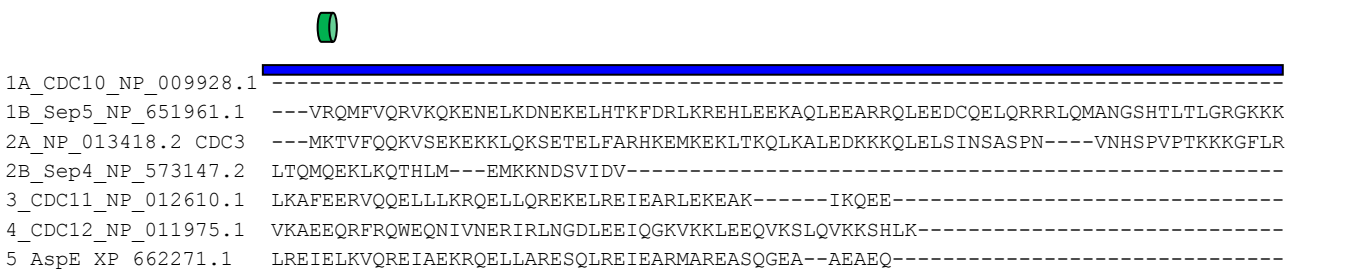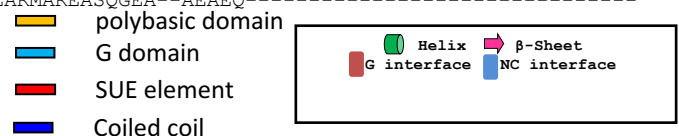

Supplement: Supplementary file 2 — Figure S1. Maximum likelihood phylogenetic analysis with RAxML software. Node values represent bootstrap support. Protein names are given for septins supported by experimental evidence. Aspergillus and Drosophila sequences used to recognize septin groups are in bold. Coiled-coil domain predictions, black representing p < 0.05 and grey p < 0.10, found to the right of names. Domain predictions for proteins longer than 600 residues have been shortened with diagonal lines. Figure S2. Bayesian phylogenetic analysis. Node values represent posterior probabilities. Protein names are shown for those septins with experimental evidence. Aspergillus and Drosophila sequences used to recognize septin groups are in bold. Coiled-coil domain prediction, black representing p < 0.05 and grey p < 0.10, shown to the right of names. Coiled-coil predictions for proteins longer than 600 residues have been shortened with diagonal lines. Figure S3. Bayesian phylogeny with jPRIME software. Topology represents maximum clade credibility tree; node values represent bootstrap support. Protein names are shown for those septins with experimental evidence. Aspergillus and Drosophila sequences used to recognize septin groups are in bold. Coiled-coil domain prediction, black representing p < 0.05 and grey p < 0.10 to the right of septin names. Coiled-coil predictions for proteins longer than 600 residues have been shortened with diagonal lines. Figure S4. Ancestral state reconstructions for presence of septin groups inferred using Mesquite with the MK1 symmetrical model. Shading of pie charts at nodes represent proportional likelihood of a node containing a member of that septin group. Statistical test showing that MK1 could not be rejected appears below state reconstructions. This test supports assuming a single rate of change for gains and losses. Figure S5. Interacting residues. A) Interacting residues found based on modelling the 5 individual crystal structures. Red or blue shading indicates the p [file 12862_2018_1297_MOESM2_ESM.zip › FigS6.Jan10.pdf]
